# Altered regulation and expression of genes by BET family of proteins in COPD patients

**DOI:** 10.1371/journal.pone.0173115

**Published:** 2017-03-01

**Authors:** Rajneesh Malhotra, Nisha Kurian, Xiao-Hong Zhou, Fanyi Jiang, Susan Monkley, Amy DeMicco, Ib G. Clausen, Göran Delgren, Goran Edenro, Miika J. Ahdesmäki, Maryam Clausen, Lisa Öberg, Elisabeth Israelsson, Graham Belfield, Outi Vaarala

**Affiliations:** 1 AstraZeneca, Respiratory, Inflammation and Autoimmunity iMed, Pepparedsleden 1, Mölndal, Sweden; 2 Thoraxtransplantation, Transplantationscentrum, Sahlgrenska Universitetssjukhuset, Göteborg, Sweden; 3 AstraZeneca, Oncology iMed, AstraZeneca, Cambridge, CB2 0RE, United Kingdom; 4 AstraZeneca, Discovery Sciences, Pepparedsleden 1, Mölndal, Sweden; 5 Clinicum, University of Helsinki, Helsinki, Finland; Pennsylvania State University, UNITED STATES

## Abstract

**Background:**

BET proteins (BRD2, BRD3, BRDT and BRD4) belong to the family of bromodomain containing proteins, which form a class of transcriptional co-regulators. BET proteins bind to acetylated lysine residues in the histones of nucleosomal chromatin and function either as co-activators or co-repressors of gene expression. An imbalance between HAT and HDAC activities resulting in hyperacetylation of histones has been identified in COPD. We hypothesized that pan-BET inhibitor (JQ1) treatment of BET protein interactions with hyperacetylated sites in the chromatin will regulate excessive activation of pro-inflammatory genes in key inflammatory drivers of alveolar macrophages (AM) in COPD.

**Methods and findings:**

Transcriptome analysis of AM from COPD patients indicated up-regulation of macrophage M1 type genes upon LPS stimulation. Pan-BET inhibitor JQ1 treatment attenuated expression of multiple genes, including pro-inflammatory cytokines and regulators of innate and adaptive immune cells. We demonstrated for the first time that JQ1 differentially modulated LPS-induced cytokine release from AM or peripheral blood mononuclear cells (PBMC) of COPD patients compared to PBMC of healthy controls. Using the BET regulated gene signature, we identified a subset of COPD patients, which we propose to benefit from BET inhibition.

**Conclusions:**

This work demonstrates that the effects of pan-BET inhibition through JQ1 treatment of inflammatory cells differs between COPD patients and healthy controls, and the expression of BET protein regulated genes is altered in COPD. These findings provide evidence of histone hyperacetylation as a mechanism driving chronic inflammatory changes in COPD.

## Introduction

COPD is a complex multifactorial disease largely associated with chronic inflammatory responses to environmental triggers such as cigarette smoke or biomass fuel particles. These irritants can drive epigenetic changes in the chromatin of immune cells, which then contribute to the dysregulation of the inflammatory responses in the human lung [1–3]. Such post-translational modifications to histone ends define the accessibility of the chromatin and with that recruitment of different coactivators or corepressors. Histone acetylation is regulated by the levels and activities of histone acetyl transferases and histone deacetylases (HDAC), and simplistically, chromatin is transcriptionally active when lysine residues on histones H3 and H4 are acetylated. Increased acetylation of histones is reported in lung biopsies obtained from COPD patients concomitant with reduced HDAC activity as measured in the peripheral lung tissue, alveolar macrophages and in bronchial biopsy specimens [4]. In agreement with reduced HDAC expression and activity, it has been shown that the acetylation of histones H2A, H2B, H3 and H4 is increased in the lungs and alveolar macrophages of COPD patients [5]. Accordingly, in a subpopulation of COPD patients, an imbalance between HAT and HDAC activities results in hyperacetylation of histones and activation of transcriptional factors that could lead to chronic inflammation associated with COPD [4].

The covalent modifications of chromatin and DNA are recognized by structurally diverse proteins that contain one or more effector modules and are termed as readers. A family of evolutionarily conserved protein containing interaction modules that recognize acetylation sites on chromatin was identified in the early 1990s in the *brahma* gene from *Drosophila melanogaster* [6]. The acetylation binding module is termed bromodomain and to date the human proteome encodes >200 proteins containing bromodomains. The BET (bromodomain and extra-terminal) proteins (BRD2, BRD3, BRDT and BRD4) belong to this family of bromodomain containing proteins (with BRDT protein expression being restricted to testis). BET proteins bind to acetylated lysine residues in the histones of nucleosomal chromatin and function either as co-activators or co-repressors of gene expression.

Yang et al. [7] reported that chronic cigarette smoke (CS) induces epigenetic/chromatin modifications resulting in the abnormal and sustained lung inflammatory response that occurs in smokers and in patients with COPD. In a murine model they showed that levels of KC, MCP-1, IL-6, and GM-CSF were significantly increased in mouse lung homogenate at both 3 days and 8 weeks of CS exposure. Furthermore, they demonstrated using ChIP sequencing in CS exposed mouse lung that pro-inflammatory gene expression was associated with increased phosphorylation/acetylation of specific histone H3 (lys9/ser10) and histone H4 (lys12) on pro-inflammatory gene promoters. Nicodeme et al. [8] reported the anti-inflammatory potential of the synthetic compound I-BET, an inhibitor of bromodomain-containing BET proteins to acetylated histones, which disrupts the formation of the chromatin complexes essential for the LPS-induced expression of inflammatory cytokines in a temporal manner (early middle and late response). These findings were further supported by Chen et al. [5]. They reported that cigarette smoke induced down-modulation of HDAC1 expression and increased H3K9 acetylation. These modifications were associated with altered expression of pro-inflammatory mediators in CS-induced rat lungs and in macrophages.

These reported observations in preclinical models and increase in histone acetylation in alveolar macrophages from COPD patients suggest a role for epigenetic pathways in chronic lung inflammation in COPD patients. To understand a role for BET family of proteins in regulation of macrophage function, we analyzed the effect of the pan-BET inhibitor JQ1, on LPS-induced gene expression in alveolar macrophages from COPD patients. We propose that JQ1 treatment modulates the interactions of BET proteins with hyperacetylated sites in the chromatin, which leads to down-regulation of the excessive activation of pro-inflammatory genes in a subset of COPD patients who show increased expression of BET regulated genes.

## Materials and methods

### Isolation and analysis of human alveolar macrophages and peripheral blood mononuclear cells

Written informed consent was obtained from an exploratory study in patients undergoing lung transplantation or resection surgery in Sahlgrenska University Hospital, Gothenburg according to protocols approved by the local ethics committee in Gothenburg (Dnr: 657–12). None of the transplant donors were from a vulnerable population and all donors or next of kin provided written informed consent that was freely given. Human alveolar macrophages were derived from these tissue lung resection or transplant tissue by flushing tissue with sterile Ca2+ Mg2+ -free PBS (Life Technologies) using a 19 gauge needle (BD). For functional assays, cells were seeded at a density of 200,000 cells per well in a 96-well tissue-culture grade flat bottom plate (Costar). Following plating, non-adherent cells were removed by copious washing with serum-free RPMI (Life Technologies) after an hour’s rest. After the final wash, the cells were incubated overnight in XVivo10 media (Lonza) supplemented with 4mM L-glutamine (Sigma) and 1% penicillin-streptomycin (Sigma).

PBMC from COPD patients or normal control subjects were isolated by density gradient separation using Lymphoprep (StemCell Technologies) using standard methodology. Isolated PBMC were seeded at a density of 200,000 cells per well in a 96-well tissue-culture grade U bottom plate (Costar) in XVivo10 media (Lonza) supplemented with 4mM L-glutamine (Sigma) and 1% penicillin-streptomycin (Sigma). The alveolar macrophages for these cytokine secretion assays were derived from lung resection from 10 patients with cancer and/or COPD patients.

AM or PBMC were incubated with a range of concentrations of the pan-BET inhibitor JQ1 for 1h at 37°C and subsequently challenged with 100ng/ml or 10ng/ml respectively of LPS from *E*. *coli* (serotype 026:B6, Sigma) for 6h or 24 h at 37°C in CO_2_ incubator. DMSO-treated cells were used as a vehicle control. Cytokine release was analyzed by standard ELISA techniques (MSD).

The viability of the AM in the presence of JQ1 was tested with WST-1 reagent (Roche). The WST-1 reagent was diluted inXVivo10 media according to the manufacturer’s instructions. The cell plate was then incubated at 37C in a 5% CO2 incubator and absorbance read after 60-90min No reduction in viability was seen with JQ1 treatment (data shown in S1 Appendix: Effect of JQ1 on viability of AM isolated from COPD patients).

### Total RNA preparation

Alveolar macrophages isolated from transplant tissue were challenged with LPS in the presence or absence of with JQ1 (10μM) as described above was used to prepare total RNA using RNeasy Mini Kit (Qiagen) according to manufacturer’s protocol. RNA integrity and concentration was assessed on the Bioanalyzer using the RNA 6000 Nano Kit (Agilent technologies).

### mRNA expression analysis–Quantitative RT-PCR

RNA was isolated from alveolar macrophages using RNeasy kits (Qiagen) according to manufacturer’s instructions. cDNA was generated using the High Capacity RNA to cDNA kit (Applied Biosystems) according to manufacturer instructions. Taqman qRT-PCR analysis was carried out using Taqman Fast Advanced mastermix (Applied Biosystems) and Taqman probes specific to cDNAs of interest. Analysis was performed using the QuantStudio 7 Flex system. Relative expression is calculated as 2^-(Ct_experimental_−CT_SDHA_). Differential expression is calculated using the DMSO +LPS and DMSO unstimulated controls to define maximum and minimum values.

### mRNA expression analysis—Next generation sequencing

Next generation sequencing (NGS) was performed on alveolar macrophages obtained from lung transplantation in COPD patients with no signs of cancer. The AM poly-mRNA fraction was purified from total RNA using the TruSeq Stranded mRNA Sample Preparation kit (Illumina). Magnetic beads with attached poly-T oligonucleotides and library preparation was performed by fragmenting mRNA molecules before copied into first strand cDNA using reverse transcriptase and random primers. Products were purified and enriched by PCR to create the final library. Quality controls (QC) were carried out at different library preparation steps using Qubit 3.0 (Invitrogen) in order to validate library quality and to determine input RNA for sequencing (15nM). Resulting library fragments were analyzed using BioAnalyzer DNA 1000 (Agilent Technologies). To facilitate cluster generation, the libraries were barcoded using adaptamers provided in the kit, according to provided protocol.

The prepared barcoded mRNA libraries were sequenced using the Illumina NextSeq 500 platform (75 bp single reads), at a sequencing depth of 10M reads in order to obtain an accurate, high-resolution view of the transcriptome.

Bcbio version 0.8.9 [9] was used for quantifying the data. STAR [10] was used within bcbio to align the data against the human hg19 assembly, yielding between 8M and 13M alignments. Gene counts were quantified using the feature Counts [11] within bcbio. CuffLinks [12] 2.2.1 was used in bcbio to quantify gene level FPKM levels. Variables with no FPKM counts in any sample were omitted from the dataset. The remaining data underwent upper quartile normalisation.

### AM transcription data analysis

DESeq2 [13] was used to identify differentially expressed genes. Six AM group comparisons were made: (i) Untreated cells vs. LPS treated for 6 hours; (ii) Untreated cells vs. LPS treated for 24 hours; (iii) LPS treated vs. LPS and JQ1 treated for 6 hours; (iv) LPS treated vs. LPS and JQ1 treated for 24 hours; (v) Untreated cells vs. JQ1 treated for 6 hours; and (vi) Untreated cells vs. JQ1 treated for 24 hours. Only protein coding genes with base mean of greater than 5 were considered. All comparisons were paired based on donor identity. Genes were classified as upregulated by LPS if they exhibited log_2_ fold change greater than 2 for untreated vs. LPS treated (at 6 or 24 hours). Genes were classified as JQ1 suppressed in the presence of LPS if they exhibited log_2_ fold change less than -2 for LPS treated vs. LPS and JQ1 treated but were excluded if they also exhibited log_2_ fold change less than -2 for untreated vs. JQ1 treated (at either 6 or 24 hours). In all cases the false discovery rate (FDR) adjusted p-value was ≤ 0.05.

### COPD expression data

To address the expression of the JQ1 modulated genes in a wider COPD population, we investigated the published gene expression data available from whole blood and sputum samples from the ECLIPSE clinical study [14]. CEL files were downloaded from Gene Expression Omnibus (GSE71220; whole blood and GSE22148, sputum) and analysed using ArrayStudio version 9 (OmicSoft,Cary, NC). The data was processed with RMA: Robust Multiarray Average [15] using a cfd-file from BrainArray [16] version 21 (hugene11st_Hs_ENSG.cdf for GSE71220 and HGU133Plus2_Hs_ENSG.cdf for GSE22148).

SVA [17] was used to identify 2 surrogate variables using the presence of statins as a variable of interest for the full model and including sex and age as adjustment variables in the null model. One significant surrogate variable was removed using the Remove Batch Effect-function in ArrayStudio. One sample was identified as an outlier (GSM1830407_S600) and was removed from further analysis.

To enable a split of responders vs non-responders, K-means clustering [18,19] was used to separate with whole blood COPD and Control subjects (only non-statin users) into two clusters. Thereafter hierarchical clustering (using correlation as the distance metrics) was used to identify a group of genes that could separate the two identified clusters. Data was normalised using robust centre scale (the row median is subtracted from each individual value and then scaled by dividing by the row median absolute deviation) for heat map generation.

GSVA: Gene set variant analysis [20] was used to test the enrichment of the gene sets within the COPD or control groups in the whole blood and sputum datasets. GSVA enrichment scores (ES) were generated using maximum difference option for both the 10 gene signature and the 83 of the 87 Group2 genes that mapped to the probes in the ECLIPSE whole blood dataset. The ES for the Control and COPD groups were compared using t-test to generate p-values for the different gene sets.

## Results

### Inflammatory genes regulated in alveolar macrophages following treatment with the pan-BET inhibitor JQ1

We performed a whole transcriptome analysis of LPS stimulated alveolar macrophages from six COPD patients using RNA-Seq to analyze the differential gene expression in response to treatment with the pan-BET inhibitor JQ1.

**Fig 1:**
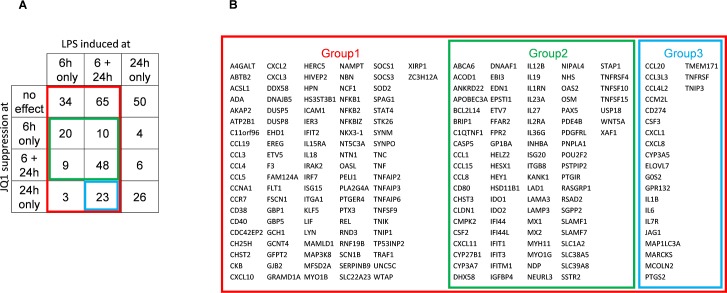
Categorisation of genes. (A) Venn diagram showing the four comparisons and the overlaps. Group 1 (red) represents the 212 genes induced by LPS at 6 hours. Group 2 (green) represents the 87 genes induced by LPS at 6 hours and inhibited following pan-BET inhibitor JQ1 treatment at 6 hours. Group 3 (blue) represents the 23 genes induced by LPS at both 6 and 24 hours and inhibited following JQ1 treatment at only 24 hours. (B) A table showing the identity (Gene Symbols) of genes in the three groups 1, 2 and 3.

Differentially expressed genes were identified for four AM group comparisons made: (i) Untreated cells vs. LPS treated for 6 hours; (ii) Untreated cells vs. LPS treated for 24 hours; (iii) LPS treated vs. LPS and JQ1 treated for 6 hours; and (iv) LPS treated vs. LPS and JQ1 treated for 24 hours (Fig 1A). Three key groups of genes were identified (Fig 1A and 1B); Group 1 contains 212 genes induced by LPS at 6 hours; Group 2 contains 87 genes induced by LPS at 6 hours and inhibited following JQ1 treatment at 6 hours (i.e. early inhibition); and Group 3 consists of 23 genes sustainably induced by LPS (i.e. at both 6 hours and 24 hours) and inhibited following JQ1 treatment at only 24 hours (i.e. late inhibition) Log_2_ fold changes ≥ 2 or ≤ -2, FDR p-value ≤ 0.05) was applied. See the supporting information for the complete list of differentially expressed genes on LPS and JQ1 treatment described in Fig 1A (S2 Appendix iBET_AM_gene_lists) and the combined list of all differentially expressed genes on treatment with LPS and JQ1 (S3 Appendix: iBET2_combined_DEG_all). The data discussed in this publication have been deposited in NCBI's Gene Expression Omnibus [21] and are accessible through GEO Series accession number GSE92532 (https://www.ncbi.nlm.nih.gov/geo/query/acc.cgi?acc=GSE92532).

We measured the RNA expression level of BET genes (BRD2, BRD3, BRD4, and the known testes specific gene BRDT) in alveolar macrophages from six COPD patients and in whole blood from the ECLIPSE COPD patients (S4 Appendix: Expression of BET genes in COPD donors from the ECLIPSE studies). We included the testes specific BRDT gene for completeness. We observed no significant changes in expression (being Log2 FC>2 at pval ≤ 0.05) for any BET protein in either study. Additionally we see no significant expression of the testes specific BRDT gene. However BRD2 was the prominent gene expressed in alveolar macrophages and is likely to be the main driver of the LPS induced gene expression in alveolar macrophages. We did not observed reduced cell viability in the presence of 10 μM JQ1 (S1 Appendix: Effect of JQ1 on viability of AM isolated from COPD patients).

**Fig 2:**
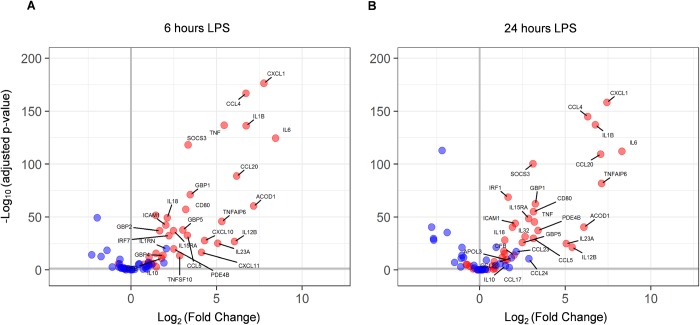
M1/M2 polarisation of LPS stimulated genes. Volcano plot of M1 (red) and M2 (blue) genes at 6 hours (A) and 24 hours (B) after LPS induction. Differential expression of unstimulated vs. LPS stimulated cells was tested using DESeq2 and the Log2 fold change (x-axis) is plotted against the–Log10 adjusted p-values (y-axis). Genes with LFC > 1.5 are labelled by name. The vertical grey lines denotes no LPS induced expression change. The horizontal grey lines shows the 0.05 adjusted p-value cut-off.

We observed that LPS treatment of alveolar macrophages induced up-regulation of detected M1 phenotype related genes (ACOD1, APOL3, IL15RA, CCL4, CCL5, CCL20, CD80, CFB, CXCL1, CXCL10, CXCL11, GBP1, GBP2, GBP4, GBP5, ICAM1, IL1B, IL6, IL12B, IL18, IL23, IL32, IRF1, IRF7, PDE4B, SOCS3,TNF, TNFAIP6, TNFSF10) over detected M2 phenotype related genes (IL10, IL1RN, CCL17, CCL22, CCL23, CCL24) across both 6 and 24 hours (Fig 2) based on previously reported M1 and M2 polarization patterns in human alveolar macrophages [22]. We compared the genes upregulated in our assays to those reported by Reynier et al, 2012 [23] in LPS induced human AMs, and found them to highly correlate. We observed a strong correlation between Reynier et al. and our study with respect to both the general pattern of genes induced by LPS treatment (84%), and specific genes associated with the M1 phenotype (92%).

**Fig 3:**
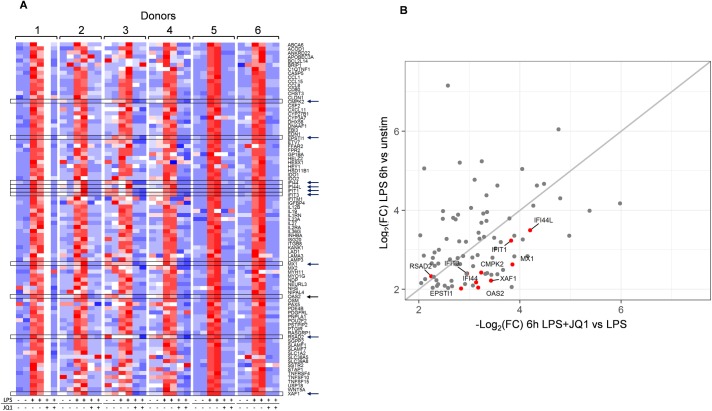
Expression of Group 2 genes at 6 hours. A: Heat map of the 87 Group 2 genes induced by LPS and suppressed following JQ1 treatment at 6 hours for each of the 6 donors. Data (TPM) was scaled and centered to a mean of 0 and standard deviation of 1 for each donor. Red color is high expression; blue color is low expression. Arrows and boxes denote the 10 genes identified as a gene signature in the ECLIPSE WB samples (discussed later). B: Plot of Log_2_ fold changes upon LPS induction (y-axis) and JQ1 treatment suppression of LPS induction (x-axis) at 6 hours. Blank (white) column (donor 1, JQ1+LPS); no sample.

Fig 3A shows a heat map of gene expression across all six donors with technical replicates for the group (2) of 87 genes related to early response to LPS and inhibition following pan-BET inhibitor JQ1 treatment. Fig 3B shows a plot of Log2 fold changes upon LPS induction at 6 hours against the JQ1 treatment suppression of LPS induction at 6 hours. These early responsive genes are predominantly associated with lymphocyte activation, differentiation and proliferation: for example CD80, EBI3, IL12-beta, IL-27, GM-CSF (CSF2), and OX40 (TNFRSF4) [24–25]. This figure demonstrates that JQ1 treatment correlates well with LPS induction for this gene set.

**Fig 4:**
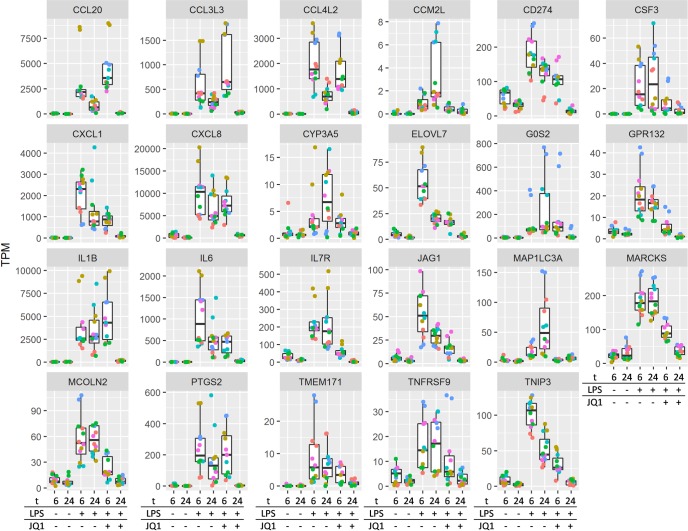
Expression profile boxplot of the 23 late inhibition genes (Group 3). Expression levels are shown in the form of transcripts per million (TPM). Each donor is represented as a different color in duplicate. Treatment groups are represented at the bottom of the plots with t = time (6; 6hours, 24; 24 hours. LPS and JQ1 indicates if these are present (+) or absent (-). Gene symbols are shown at the top of each plot.

Some genes (group 3) were not significantly down-regulated by the pan-BET inhibitor JQ1 at 6h, but their expression levels were reduced to the background level at 24h in the presence of JQ1 (Fig 4), and include genes involved in the inflammatory response and chemotaxis; exemplified here by chemokine genes CCL20, CCL3L3, CCL4L2, CXCL1, CXCL8, interleukin genes IL1B, IL6, IL7R.

### Inhibition of IL-6 secretion following JQ1 treatment in alveolar macrophages from COPD patients

**Fig 5:**
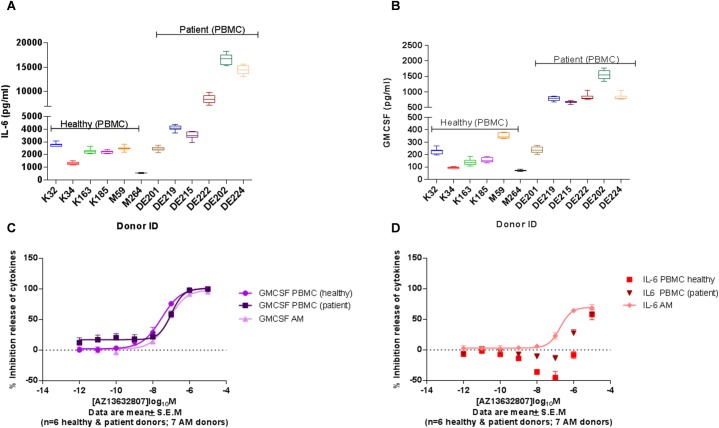
Effect of pan-BET inhibitor JQ1 treatment on COPD patient derived lung resident alveolar macrophages and Peripheral Blood Mononuclear Cells (PBMC). LPS-induced secretion of IL-6 (A) and GM-CSF (B) from from COPD patients and normal controls. PBMC from COPD patients were more sensitive to LPS stimulation producing higher levels of cytokines. JQ1 (AZ13632807) treatment lead to a more potent inhibition of LPS-induced IL-6 secretion in alveolar macrophages from COPD patients than observed in PBMC (D), whereas inhibition of GM-CSF was identical in the three cellular systems (C).

As shown above, the up-regulation of GM-CSF (CSF2) and IL6 gene expressions were inhibited following JQ1 treatment at 6 and 24 hours, respectively. Thus, we decided to use GM-CSF and IL-6 as the read-outs when comparing the effect of JQ1 treatment on LPS stimulated peripheral blood mononuclear cells and alveolar macrophages derived from COPD patients and normal controls. First, we found enhanced GM-CSF and IL-6 secretion as response to LPS in PBMCs from COPD patients when compared to normal controls (Fig 5A and 5B). JQ1 is a potent pan-BET inhibitor with a cellular potency EC50 value of 150nM. We observed differential inhibition potencies between GM-CSF and IL-6 protein production following JQ1 treatment (Fig 5C and 5D). We demonstrated effective inhibition of GM-CSF response following JQ1 treatment in PBMCs from both patients and normal controls as well as in AM from COPD patients with an EC50 value for the inhibition of 150nM, which indicates that GM-CSF secretion is regulated by BET proteins. However, the IL-6 response was effectively inhibited following JQ1 treatment only in AM and PBMCs from COPD patients with approximately 50% inhibition and 25% inhibition at 1μM in alveolar macrophages and PBMC from COPD patients, respectively, whereas no inhibition was seen in PMBC from normal controls (Fig 5D). Thus, BET mediated regulation of IL-6 response was only evident in COPD samples suggesting a difference in the histone acetylation between COPD derived lung and peripheral inflammatory cells vs peripheral blood inflammatory cells from healthy individuals. To our knowledge, this is the first demonstration that treatment with the pan-BET inhibitor JQ1 differentially regulates LPS-induced cytokine release from AM or peripheral blood mononuclear cells (PBMC) of COPD patients compared to healthy controls. Belinka et al. [26] reported JQ1 treatment mediated modulation of gene expression in mouse bone marrow derived macrophages. Belinka and co-workers reported the role of individual BET proteins and pan-BET JQ1 treatment in LPS mediated cytokine release in rodent models and rodent cell lines, but in human PBMC or alveolar macrophages we observed a different profile.

### Analysis of BET regulated genes in COPD patients

To address the expression of pan-BET inhibitor JQ1 modulated genes in a wider COPD population, we investigated the published gene expression data available from whole blood and sputum samples from the ECLIPSE clinical study [14]. ECLIPSE is a longitudinal study conducted by GSK in COPD subjects and a small number of smoking control subjects that were followed regularly for 3 years, including 3 chest CT scans and expression of approximately 12000 genes in sputum and PBMC is reported in the databases.

We investigated a subset from this study consisting of 405 COPD patients and 44 normal control patients. We saw no significant expression of BRDT or BTD3, and no significant change in expression of BRD2 and BRD4 in the ECLIPSE samples (S4 Appendix: Expression of BET genes in COPD donors from the ECLIPSE studies).

**Fig 6:**
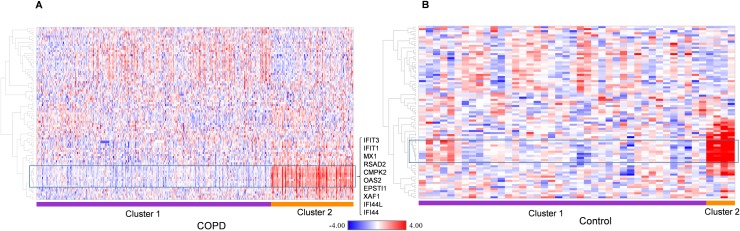
ECLIPSE COPD and normal controls K-means clustering using the early JQ1 treatment gene set. Gene expression heat maps of (A): ECLIPSE COPD samples and (B): ECLIPSE normal control samples following k-means clustering. Membership of samples to one of the two clusters is shown below each heat map (purple; cluster 1, orange; cluster 2). Data in each figure are normalized by subtracting the row median then scaling by the row median absolute deviation. Red color denotes high expression; blue color denotes low expression. 83 of the 87 early JQ1 treatment genes are present in the ECLIPSE study. The box highlights the 10 gene “signature” that shows strong association with cluster 2 of the ECLIPSE COPD samples and these are listed to the right. This same set of genes are also highlighted (box) on the ECLIPSE control heat map.

We applied the K-means clustering approach [18, 19] to investigate whether the early (6h) JQ1 treatment set of 87 genes contained a subset of genes that are highly variable across the COPD population. We discovered 83 of the 87 early JQ1 treatment genes are present in the ECLIPSE study. Fig 6 shows gene expression heat maps revealing two distinct clustered groups in both COPD and control populations (cluster 1 and 2). A 10 gene subset of the 83 genes show a strong association with cluster 2 of the ECLIPSE COPD samples (Fig 6A). This approach was then applied to the normal control group of 44 samples, and the same set of 10 genes showed a strong association with a smaller cluster in the ECLIPSE controls (Fig 6B). Hereafter this subset of 10 genes (CMPK2, EPSTI1, IFI44, IFI44L, IFIT1, IFIT3, MX1, OAS2, RSAD2 and XAF1) will be described as the “signature genes”.

**Fig 7:**
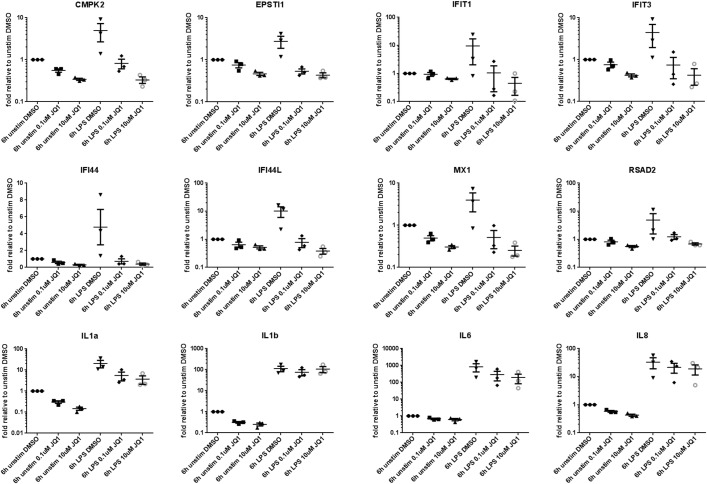
Quantitative PCR validation of selected genes identified by NGS. Effect of JQ1 treatment on LPS induced expression of signature genes and cytokine control genes in AM from COPD patients. JQ1 treatment lead to inhibited expression of 8 signature genes (CMPK2, EPSTI1, IFI44, IFI44L, IFIT1, IFIT3, MX1 and RSAD2) but had a partial effect on cytokines (IL1a, IL1b, IL6 and IL8) not inhibited at 6h in NGS studies.

To further validate the genes modulated following JQ1 treatment in alveolar macrophages, we analyzed their gene expression by TAQMAN in alveolar macrophages from three COPD patients. In these experiments AM derived from COPD patients were stimulated with LPS in the presence or absence of JQ1 for 6hrs as described above and change in expression of 8 out of 10 signature genes (XAF1 and OAS2 were not analyzed for technical reasons) and 4 cytokines was analyzed by RT-PCR (Fig 7). Expression of all the 12 genes was increased with LPS treatment, and the expression of signature genes were effectively decreased to untreated levels in the presence of JQ1, whereas expression of pro-inflammatory genes remained unchanged or only modestly reduced with JQ1 treatment. These results confirms the specific effect of JQ1 treatment on the inhibition of signature genes.

**Fig 8:**
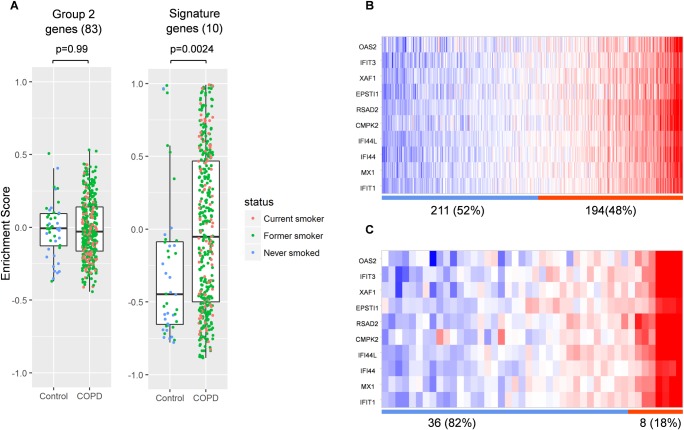
Subgroups of ECLIPSE WB COPD patients characterised by the 10 gene signature. A: Plots of gene set enrichment scores generated using either the entire set of early response genes (left) or the 10 gene signature (right) with p-values (t-test, COPD vs control). B Heat maps showing the distribution of the 10 gene signature across samples split into 2 groups based on enrichment score for either the COPD samples (B) or Control samples (C). Numbers and percentage of samples in each group are below the heat maps. Blue bar; negative enrichment score, orange bar; positive enrichment score.

To further validate and better understand the significance of the 10 gene signature we performed gene set variation analysis (GSVA) in the ECLIPSE WB COPD patients. Fig 8A shows plots of gene set enrichment scores generated using either the complete set of early response genes (group 2 in Fig 1) or the 10 gene signature (defined in Fig 6). This demonstrates a significant enrichment (p = 0.0024) in COPD WB only for the signature genes. Fig 8 also shows the distribution of the 10 gene signature across samples split into 2 groups based on enrichment score for either the COPD samples (Fig 8B) or Control samples (Fig 8C). The clustering reveals a distinct separation of the signature in disease over normal populations, with 48% of COPD patients being enriched for the signature, compared with 18% control patients.

**Fig 9:**
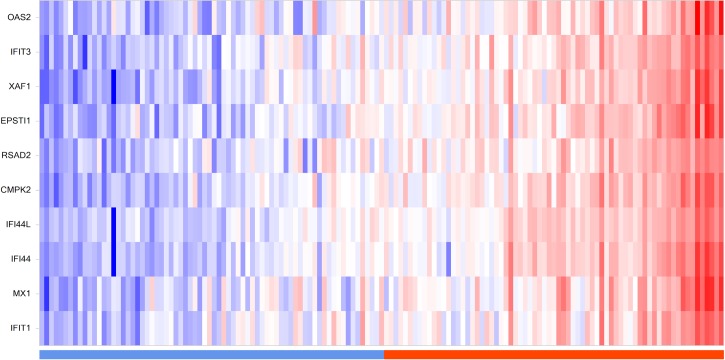
Signature distribution in ECLIPSE sputum samples. Heat map of the ECLIPSE sputum data for the 10 signature genes. Samples are ordered according to the enrichment scores generated by GSVA with the 10 gene signature. The value distribution is shown in the top panel. Data was normalized by subtracting the row median then scaling by the row median absolute deviation. Red color denotes high expression; blue color denotes low expression. Bar below indicates GSVA enrichment score for these samples as either negative (blue) or positive (orange).

We also observed that COPD patients from ECLIPSE show a similar enrichment for the 10 gene signature in sputum samples (Fig 9). The study includes no sputum samples from healthy controls and the analysis platforms for sputum and whole blood are different, so a comparison to healthy controls cannot be made. However, the differential expression level of BET protein regulated genes (following JQ1 treatment) in blood and sputum could define a segment of COPD patients with aberrant regulation of the gene set associated with histone hyperacetylation, which could be used as a signature for the patient stratification in intervention studies targeting BET proteins.

## Discussion

To increase our understanding of the genes regulated by the pan-BET inhibitor JQ1 and thus associated with histone hyperacetylation in COPD lung derived inflammatory cells, we performed a global gene expression approach on LPS stimulated alveolar macrophages from six COPD donors, and looked at the impact on gene expression of JQ1 treatment. Our findings are in agreement with earlier studies reporting global gene expression following LPS stimulation of human macrophages [23, 27]. In a comparable human alveolar macrophage study [23] we see a strong correlation with respect to both the general pattern of genes induced by LPS treatment (84%), and specific genes associated with the M1 phenotype (92%).

In the analysis of LPS-induced genes inhibited following JQ1 treatment, we identified two sets of genes, early and late response genes, The major enriched group of genes in the early response genes group, i.e. genes inhibited at 6h, are known to be involved in cell activation, differentiation and proliferation: for example CD80, EBI3, IL12-beta, IL27A, GM-CSF(CSF2), and OX40 (TNFRSF4). IL-27 consists of EBI3, an IL-12p40-related protein, and p28, an IL-12p35-related polypeptide. IL-27 is an early product of activated antigen-presenting cells and regulates T cell development and proliferation. It also synergizes with IL-12 to trigger IFN-gamma production [23]. TNFRSF4 (OX40) and CD80 regulate the T cell activation and antigen presentation.

Chan et al. [24] found that I-BET151 selectively suppresses expression of a subset of IFN-β-induced interferon signaling genes, among these IFIT1 and IFIT2. We identify IFIT1 and other interferon signaling genes to form a group of early JQ1 treatment genes represented by genes IFIT1, IFIT3, OAS2, IFI44, MX1 and MX2, which may play a role in inducing pro-inflammatory cytokines as part of an innate immune response.

The second group of genes (late response) inhibited at 24 h are genes associated with inflammatory response, e.g. IL1B, IL6, IL7R and chemotaxis chemokines, e.g. CCL20, CCL3L3, CCL4L2, CXCL1, CXCL8. Interestingly, all these are involved with inflammatory pathology of the COPD and are common to the inflammatory pathology of several inflammatory diseases other than COPD. It is very likely that the early response genes regulate the expression of some of these genes. To demonstrate the effect of the BET inhibition on the inflammatory response at the protein level, we measured GM-CSF and IL-6 secreted by LPS stimulated alveolar macrophages or PBMC from COPD patients and PBMC from normal controls. Treatment with the pan-BET inhibitor JQ1 lead to potent inhibition of LPS-induced GM-CSF release from alveolar macrophages and PBMC from COPD patients. These results indicate that inhibition of GM-CSF following JQ1 treatment was complete and independent of the cell type, i.e. the potency of JQ1 for inhibition of GM-CSF was similar in alveolar macrophages and PBMC. However, IL-6 was only partially inhibited and the EC_50_ value for IL-6 was 10x less than for inhibition of GM-CSF in alveolar macrophages. Furthermore, the potency of JQ1 treatment inhibition of IL-6 in PBMC from COPD patients was almost 100x less than inhibition of GM-CSF. At the concentration of 1μM, no inhibition of IL-6 following JQ1 treatment was observed in PBMC from normal controls. To our knowledge, the effect of JQ1 treatment on human PBMC or alveolar macrophages has not been previously reported. On the other hand, the effect of JQ1 treatment is reported to have a potent effect on inhibition of IL-6 and TNF-alpha in both mouse primary bone marrow derived macrophages and human cancerous cell lines where epigenetics is likely to play a role in modulating the cellular function [26, 28]. Furthermore we observed that a significant proportion (>30%) of LPS-induced genes inhibited following JQ1 treatment are misregulated in a GM-CSF KO mouse [29]. Altogether, these results suggest that IL-6 inhibition may be only sensitive to JQ1 treatment mediated inhibition in the cells in which the epigenetic changes are present, such as in alveolar macrophages from COPD patients. The gene expression data support the protein data, where we observed a partial inhibition of IL-6 protein secretion. We propose that alveolar macrophages from COPD have an excessive histone acetylation, which results in an overexpression of genes controlled by BET proteins.

As expression of the early response genes is directly regulated by the BET family of proteins and is independent of cell type, we hypothesised that these genes could be valuable in patient stratification to define a subpopulation of COPD patients more likely to respond to BET inhibition. To further understand the relevance of this signature in COPD patients we analysed gene expression data from the ECLIPSE study [14] made public by GlaxoSmithKline. Analysis of the expression of 10 genes—CMPK2, EPSTI1, IFI44, IFI44L, IFIT1, IFIT3, MX1, OAS2, RSAD2 and XAF1—in the COPD samples compared to samples from normal controls showed a statistically significant difference in the expression level distribution between COPD patients and normal controls. According to our analysis, a subset of COPD patients with increased expression of these signature genes at a specified interval of signature expression are likely to show maximum response to BET inhibition. A future controlled study with matched numbers of COPD and normal control samples should be made to confirm this observation. A major limitation to the study is lack of healthy smoking controls, but it is difficult to obtain sufficient amount tissue samples from the healthy patients thus limiting the scope of the study. Another potential limitation of the study is the very high concentration of pan-BET inhibitor JQ1 used in the transcriptome analysis. We chose this as we were primarily interested in identifying maximally regulated genes following pan-BET inhibition and genes regulated by JQ1 were effectively inhibited to the pre-LPS treatment basal level. Importantly we did not observe cell toxicity with 10μM JQ1 (S1 Appendix: Effect of JQ1 on viability of AM isolated from COPD patients). Furthermore in the incidences where we performed JQ1 treatments at lower doses (Fig 5 and Fig 7) we see approximately equivalent results to the higher dose i.e. GMCSF was inhibited in the PBMC cytokine secretion assay at EC50 value of approximately 150nM (Fig 5) and AMs treated with 100nM JQ1 still displayed potent inhibition of the signature genes at 6h. Finally it is important to consider that whilst the gene expression changes observed following treatment with JQ1 was regulated by BET proteins, the experimental design meant it was not possible to determine which specific BET protein or combination of BET proteins is targeted by JQ1. However we looked at the RNA expression level of BET genes (BRD2, BRD3 and BRD4) in alveolar macrophages from six COPD patients and in whole blood from the ECLIPSE COPD patients (S4 Appendix: Expression of BET genes in COPD donors from the ECLIPSE studies). Whilst we observed no significant changes in expression (being Log2 FC>2 at pval ≤ 0.05) for any BET protein in either study, we note that BRD2 was the prominent gene expressed in alveolar macrophages and is likely to be the main driver of the LPS induced gene expression in alveolar macrophages.

It should be emphasised that the effect of pan-BET inhibitor, such as JQ1, is a net-effect of the inhibition of different BET proteins, which can affect distinct transcriptional pathways by activation or repression of different genes, as demonstrated earlier in T-cells by Banerjee et al. [30] and in insulin secreting beta-cell line, reported by Deeney et al. [31]. The need to develop selective inhibitors to each BET proteins to avoid treatment adverse effects in the clinics has also been highlighted by Andrieu et al. [32]. In summary, we identified an early phase gene set modulated through JQ1 mediated pan-BET inhibition in alveolar macrophages from COPD patients. We show that the BET family of proteins regulate the expression of genes involved in T cell regulation and innate pathways. The gene pool identified to be regulated by BET proteins was differentially expressed in inflammatory cells from COPD patients and healthy controls, and could thus be used for the identification of the patients who benefit from BET inhibition as a therapy. Altogether, these findings support the role of histone hyperacetylation as an epigenetic factor contributing to the pathology of COPD.

Further research is, however, needed to understand the diverse functions of BET proteins and to develop better and more selective drug candidates.

## Supporting information

S1 Appendix Effect of JQ1 on viability of AM isolated from COPD patients. To rule out potential cytotoxic effect of JQ1 at high concentration of JQ1, we performed cell viability assay in AM from multiple COPD donors. In our assays, LPS-induced increased viability of alveolar macrophages and this increase was reduced to background (cell viability in the absence of LPS) in the presence of 10 μM JQ1. At this concentration JQ1 is reported to completely block the BRD/Histone interaction and is selective for other bromodomain containing proteins, kinases and 7TM targets [17].
(TIF)

S2 Appendix iBET_AM_gene_lists. A table containing a complete list of significantly differentially expressed genes on LPS and JQ1 treatment described in Fig 1B.
(XLSX)

S3 Appendix iBET2_combined_DEG_all. A table containing a combined NGS values for all differentially expressed genes on treatment with LPS and JQ1.
(XLSX)

S4 Appendix Expression of BET genes in COPD donors from the ECLIPSE studies. S4 Appendix A: Expression of BET proteins BRD2, BRD3, BRD4 across the 6 COPD alveolar macrophage donors. Each donor is represented as a different color. There are no significant changes in expression (significance defined by a Log2 Fold Change(FC) >2 at a p val < 0.05) for any BET protein. BRDT was not expressed in alveolar macrophages. S4 Appendix B: BRD2 and BRD4 expression in the ECLIPSE whole blood samples. Blue box are the control samples and the COPD samples are in the pink box. BRD3 and BRDT data not were not available.
(TIF)
