# Correction: Altered regulation and expression of genes by BET family of proteins in COPD patients

**DOI:** 10.1371/journal.pone.0175997

**Published:** 2017-04-12

**Authors:** Rajneesh Malhotra, Nisha Kurian, Xiao-Hong Zhou, Fanyi Jiang, Susan Monkley, Amy DeMicco, Ib G. Clausen, Göran Dellgren, Goran Edenro, Miika J. Ahdesmäki, Maryam Clausen, Lisa Öberg, Elisabeth Israelsson, Graham Belfield, Outi Vaarala

The eighth author's name is spelled incorrectly. The correct name is: Göran Dellgren.
