# Supplementary figures and images for: Altered regulation and expression of genes by BET family of proteins in COPD patients
Source: PLoS One. 2017 Mar 1;12(3):e0173115. doi: 10.1371/journal.pone.0173115 (PMC5332090; doi:10.1371/journal.pone.0173115)

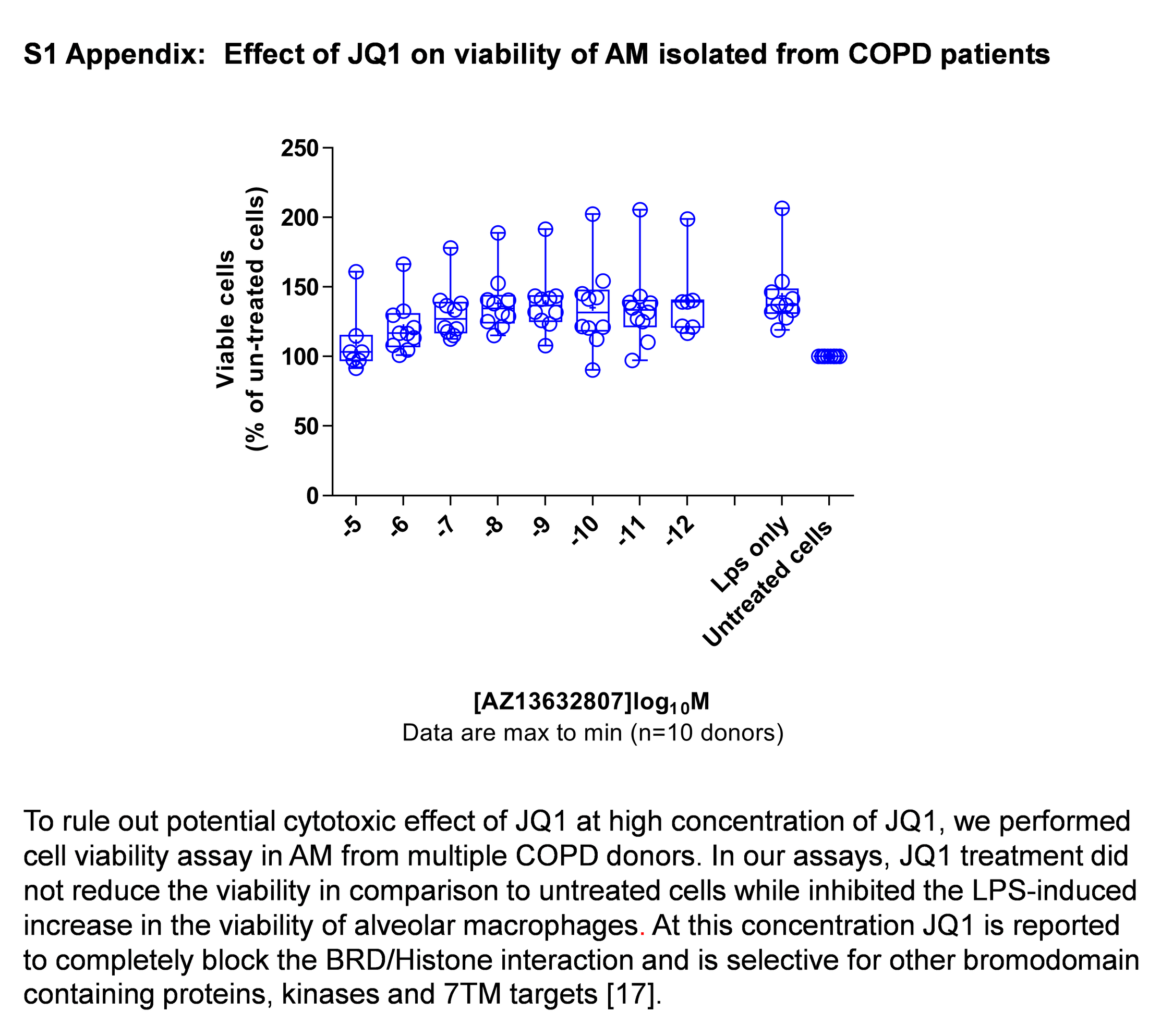

Supplement: S1 Appendix — To rule out potential cytotoxic effect of JQ1 at high concentration of JQ1, we performed cell viability assay in AM from multiple COPD donors. In our assays, LPS-induced increased viability of alveolar macrophages and this increase was reduced to background (cell viability in the absence of LPS) in the presence of 10 μM JQ1. At this concentration JQ1 is reported to completely block the BRD/Histone interaction and is selective for other bromodomain containing proteins, kinases and 7TM targets [17]. (TIF) [file pone.0173115.s001.tif]

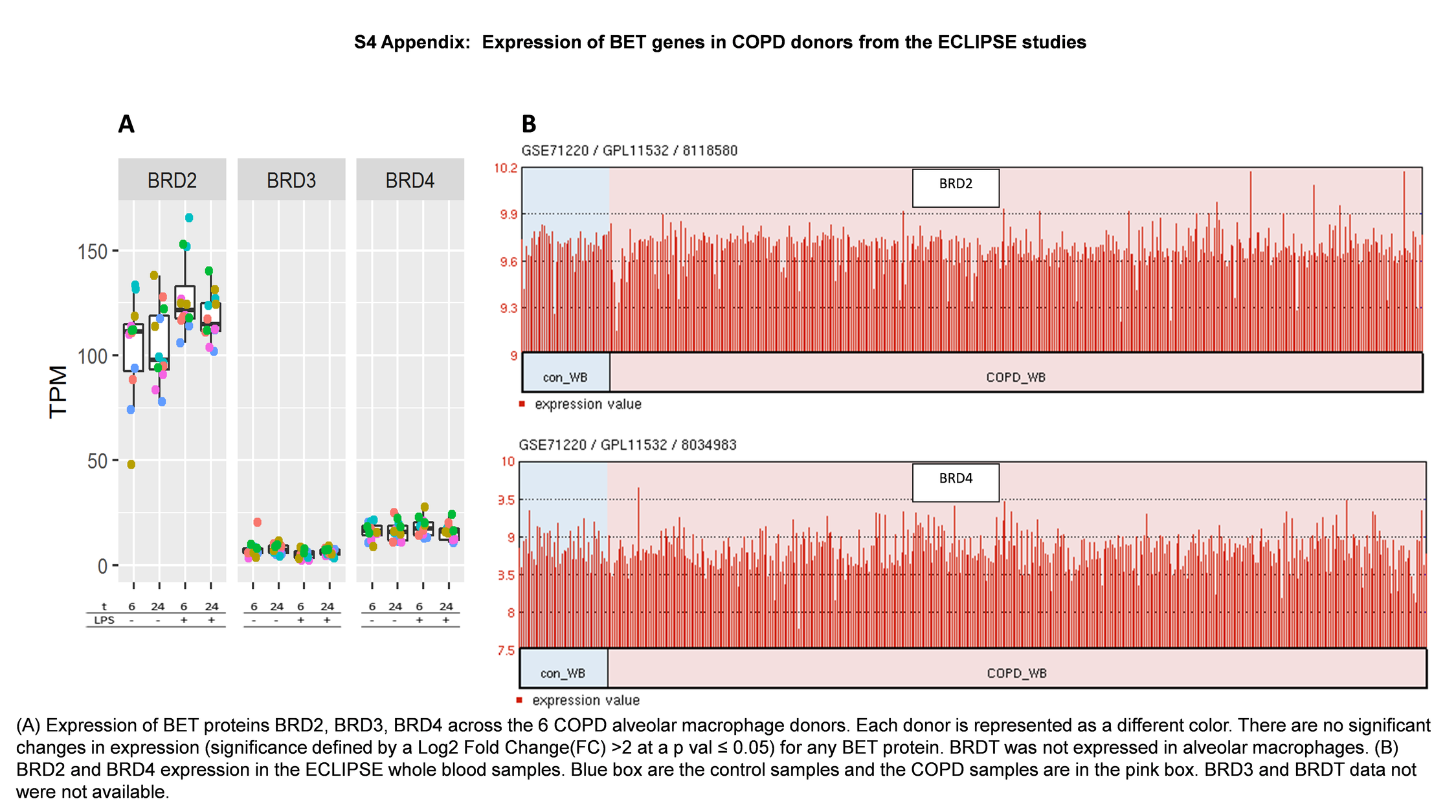

Supplement: S4 Appendix — A: Expression of BET proteins BRD2, BRD3, BRD4 across the 6 COPD alveolar macrophage donors. Each donor is represented as a different color. There are no significant changes in expression (significance defined by a Log2 Fold Change(FC) >2 at a p val < 0.05) for any BET protein. BRDT was not expressed in alveolar macrophages. S4 Appendix B: BRD2 and BRD4 expression in the ECLIPSE whole blood samples. Blue box are the control samples and the COPD samples are in the pink box. BRD3 and BRDT data not were not available. (TIF) [file pone.0173115.s004.tif]
